# Preamplification with dUTP and Cod UNG Enables Elimination of Contaminating Amplicons

**DOI:** 10.3390/ijms19103185

**Published:** 2018-10-16

**Authors:** Daniel Andersson, David Svec, Cathrine Pedersen, Jørn Remi Henriksen, Anders Ståhlberg

**Affiliations:** 1 Sahlgrenska Cancer Center, Department of Pathology and Genetics, Sahlgrenska Academy at University of Gothenburg, Box 425, 40530 Gothenburg, Sweden; daniel.andersson.3@gu.se (D.A.); david.svec@ibt.cas.cz (D.S.); 2 Institute of Biotechnology of the Czech Academy of Sciences, BIOCEV, Prumyslová 595, 25250 Vestec u Prahy, Czech Republic; 3 ArcticZymes AS, Sykehusveien 23, 9019 Tromsø, Norway; cap@arcticzymes.com (C.P.); jrh@arcticzymes.com (J.R.H.); 4 Wallenberg Centre for Molecular and Translational Medicine, University of Gothenburg, 40530 Gothenburg, Sweden; 5 Department of Clinical Pathology and Genetics, Sahlgrenska University Hospital, 41345 Gothenburg, Sweden

**Keywords:** Cod UNG, contamination, dUTP, preamplification, qPCR, single-cell analysis

## Abstract

Analyzing rare DNA and RNA molecules in limited sample sizes, such as liquid biopsies and single cells, often requires preamplification, which makes downstream analyses particularly sensitive to polymerase chain reaction (PCR) generated contamination. Herein, we assessed the feasibility of performing Cod uracil-DNA *N*-glycosylase (Cod UNG) treatment in combination with targeted preamplification, using deoxyuridine triphosphate (dUTP) to eliminate carry-over DNA. Cod UNG can be completely and irreversibly heat inactivated, a prerequisite in preamplification methods, where any loss of amplicons is detrimental to subsequent quantification. Using 96 target assays and quantitative real-time PCR, we show that replacement of deoxythymidine triphosphate (dTTP) with dUTP in the preamplification reaction mix results in comparable dynamic range, reproducibility, and sensitivity. Moreover, Cod UNG essentially removes all uracil-containing template of most assays, regardless of initial concentration, without affecting downstream analyses. Finally, we demonstrate that the use of Cod UNG and dUTP in targeted preamplification can easily be included in the workflow for single-cell gene expression profiling. In summary, Cod UNG treatment in combination with targeted preamplification using dUTP provides a simple and efficient solution to eliminate carry-over contamination and the generation of false positives and inaccurate quantification.

## 1. Introduction

Today, many clinical and scientific studies are dependent on the detection and quantification of rare analytes in limited sample sizes, such as liquid biopsies, cytological aspirates, and individual cells. The analysis of DNA and RNA is typically performed using quantitative real-time polymerase chain reaction (qPCR), digital polymerase chain reaction (dPCR), and next-generation sequencing (NGS). Preamplification is required to enable analysis of multiple targets and minimize the variability introduced by allocating few molecules among several individual reactions [1,2]. Preamplification is successfully applied in numerous applications, such as single-cell transcriptome [3,4,5,6,7,8,9,10], genome [10,11], proteome [6], and epigenetic [12] studies, as well as analysis of circulating cell-free DNA in liquid biopsies [13,14,15,16].

Many preamplification strategies are PCR-based and either target-specific [7,17] or global, targeting all molecules indiscriminately [9,18]. Target-specific preamplification is usually preferred in qPCR- and dPCR-based workflows, since assays used for downstream quantification can also be used in the preamplification step. The goal of preamplification is to generate sufficient amount of target molecules to allow singleplex quantification of multiple targets in a reproducible, specific, and sensitive manner. As this may require target sequences to be multiplied several orders of magnitude, subsequent sample handling becomes a potential contamination hazard, where highly concentrated PCR products potentially cause contamination of downstream reactions, like qPCR, dPCR, and NGS.

In PCR, deoxythymidine triphosphate (dTTP) can be replaced with deoxyuridine triphosphate (dUTP). Before PCR is initiated, the use of uracil-DNA *N*-glycosylase (UNG) will degrade any uracil-containing PCR products, i.e., eliminate carry-over contamination [19]. Consequently, only thymine-containing target DNA derived from the original biological sample can be amplified. However, the inability to completely inactive conventional UNG [20,21] makes it unsuitable to workflows involving preamplification, as any loss of amplicons will cause inaccurate quantification in downstream analyses. Cod UNG (Cod uracil-DNA *N*-glycosylase), derived from the Atlantic cod (*Gadus morhua*), is produced in a recombinant *E. coli* strain containing a modified Cod UNG gene that can be completely and irreversibly heat inactivated.

**Figure 1:**
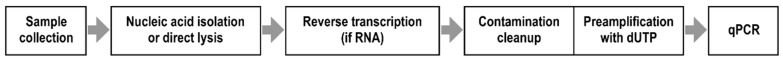
An experimental workflow. Contamination cleanup with Cod uracil-DNA N-glycosylase (Cod UNG) and preamplification can be performed separately or as one combined step. In this study, contamination cleanup was performed together with the preamplification step. Samples were UNG treated by adding Cod UNG to the preamplification master mix and by subsequently incubating the samples for 5 min at room temperature before initiating preamplification. The pre-denaturation step at 95 °C for 10 min that activate the polymerase also irreversibly inactivate Cod UNG. dUTP: deoxyuridine triphosphate; qPCR: quantitative real-time polymerase chain reaction.

Here, we studied the feasibility of performing target-specific preamplification using dUTP and Cod UNG for contamination cleanup (Figure 1). We further show how Cod UNG treatment in combination with preamplification using dUTP can be used for reliable quantification of rare molecules even in the presence of PCR generated contamination.

## 2. Results

### 2.1. Preamplification Using Uracil Is Efficient, Reproducible, and Sensitive

**Figure 2:**
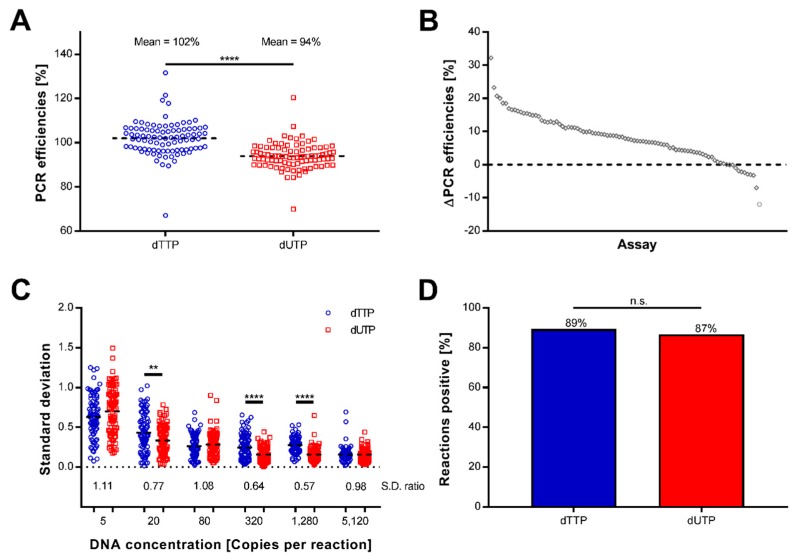
Targeted preamplification with deoxythymidine triphosphate (dTTP) and deoxyuridine triphosphate (dUTP). (**A**) Amplification efficiencies using dTTP and dUTP. Overall amplification efficiency of preamplification and quantitative real-time polymerase chain reaction (qPCR) was estimated using DNA standards. Each circle/square represents an individual assay. Dashed lines correspond to average polymerase chain reaction (PCR) efficiency. Difference in average PCR efficiency was tested using two-tailed Wilcoxon matched-pairs signed rank test (*n* = 91 assays). **** *p* < 0.0001. (**B**) Amplification efficiency variability. Difference in PCR efficiencies (E) calculated as E_dTTP_ − E_dUTP_. Each circle represents an individual assay. Dashed line corresponds to equal PCR efficiencies (*n* = 91 assays). (**C**) Amplification reproducibility. Standard deviation at six standard curve concentrations for each assay is shown. Dashed lines correspond to average standard deviation (S.D.). S.D. ratio is calculated as the ratio between average S.D._dUTP_ and average S.D._dTTP_. Difference in average standard deviation was tested using two-tailed Wilcoxon matched-pairs signed rank test (*n* = 91 assays, each concentration was analyzed as triplicate). ** *p* < 0.01; **** *p* < 0.0001. (**D**) Amplification sensitivity. Sensitivity was calculated as the percentage of positive preamplification reactions using 5 DNA standard molecules. Difference in sensitivity was tested with two-tailed Fisher’s exact test (*n* = 91 assays). n.s.: not significant.

To test the effect of replacing thymine with uracil in preamplification, we performed target-specific preamplification using either dTTP or dUTP. To evaluate amplification efficiency, reproducibility, and sensitivity, we applied high-throughput qPCR (Figure 1). The overall amplification efficiency of preamplification and qPCR was determined using absolute DNA standards generated with dTTP, in the range 5 to 5120 molecules per reaction, for 96 individually optimized assays. A total of five assays failed in the preamplification or following high-throughput qPCR and were excluded from further analysis (Table S1). The average efficiency was significantly higher using dTTP (102%) compared with dUTP (94%) for the 91 assays (*p* < 0.0001) (Figure 2A and Table S1). Moreover, 80 of 91 assays showed higher PCR efficiency using dTTP (Figure 2B), where *E2F1*, *BCL2L1*, and *CDKN2C* showed the highest differences in comparison (32%, 23%, and 21%, respectively). The use of dUTP resulted in higher efficiencies for eleven assays compared to dTTP, where *GTSE1*, *CDK7*, and *IL6ST*, displayed the highest differences (12%, 7.3%, and 3.2%, respectively, Table S1). The use of dUTP displayed improved reproducibility (*p* < 0.05) for three of the six concentrations tested (Figure 2C). As expected, the variability increased with decreasing molecule numbers using both dTTP and dUTP. To investigate whether the sensitivity to amplify few template molecules was affected, we compared the number of replicates positive at the lowest template concentration. No difference was observed between using dTTP and dUTP in preamplification (*p* > 0.05, Figure 2D). In conclusion, using dUTP instead of dTTP in target-specific preamplification results in decreased amplification efficiency, increased reproducibility, and similar sensitivity.

### 2.2. Cod UNG Is Compatible with Preamplification and Removes Carry-Over Contamination

**Figure 3:**
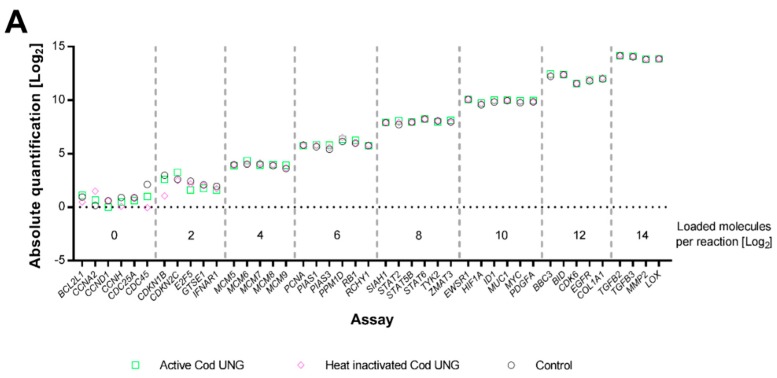

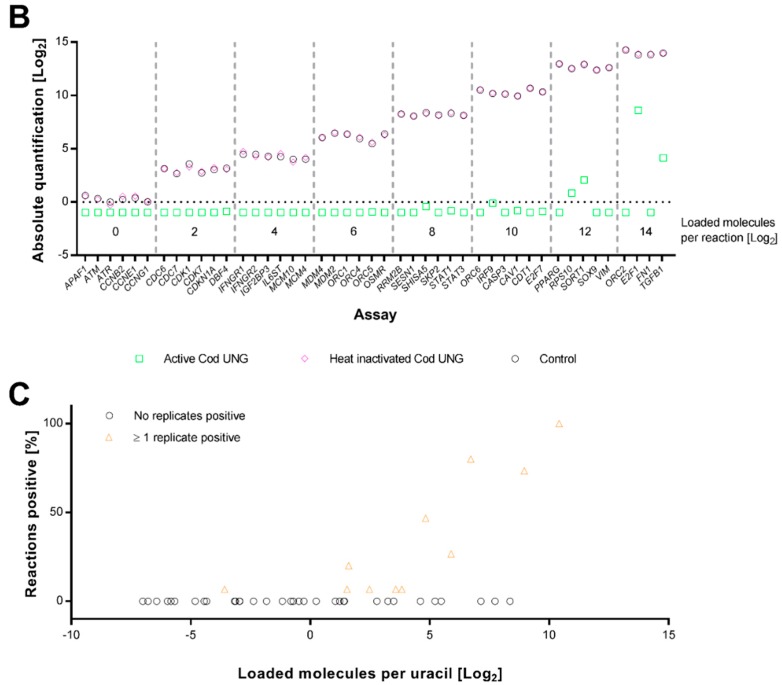
The effect of Cod uracil-DNA N-glycosylase (Cod UNG) treatment on thymine- and uracil-containing DNA standards. (**A**) Cod UNG inhibition test: 43 individual thymine-containing DNA standards were successfully preamplified and analyzed by quantitative real-time polymerase chain reaction (qPCR). Four to six different assays were amplified at each concentration, ranging from 1 to 16,384 molecules per reaction. Prior to preamplification, samples were treated with Cod UNG (green squares), heat inactivated Cod UNG (purple diamonds), or water (control, black circles). Numbers on the x-axis correspond to the number of molecules loaded per reaction, whereas numbers on the y-axis correspond to the number of molecules measured per reaction. The difference in absolute quantities was assessed using the nonparametric Friedman test followed by Dunn’s multiple comparisons test, comparing Cod UNG treatment and heat inactivated Cod UNG treatment with the control group. No statistical significance between Cod UNG and water, nor between heat inactivated Cod UNG and control, was observed (*n* = 15 reactions per treatment). (**B**) Cod UNG efficiency test: 45 individual uracil-containing DNA standards were successfully preamplified and analyzed by qPCR. 4 to 6 different assays were amplified at each concentration, ranging 1 to 16,384 molecules per reaction. Prior to preamplification, samples were treated with Cod UNG (green squares), heat inactivated Cod UNG (purple diamonds) or water (control, black circles). Numbers on the x-axis correspond to the number of molecules loaded per reaction. Numbers on the y-axis correspond to the number of molecules measured per reaction. The difference in absolute quantities was assessed using the nonparametric Friedman test, followed by Dunn’s multiple comparisons test, comparing Cod UNG and heat inactivated Cod UNG treatment with the control group. No statistical difference between heat inactivated Cod UNG and control was observed, whereas Cod UNG displayed significantly lower yield than control (*p* < 0.0001, *n* = 15 reactions per treatment). (**C**) Uracil-containing DNA standards positive after Cod UNG treatment. Percentage of positive samples plotted against number of loaded molecules per uracil, the last calculated at total number of uraciles in the sequence between the primers. For example, the *E2F1* assay was loaded with 16,384 DNA standard molecules and the amplicon contained 12 uraciles, i.e., 16,384/12 = 1365 loaded molecules per uracil. Each black circle and orange triangle corresponds to one assay (*n* = 15 replicates per assay).

We also evaluated how efficiently Cod UNG degrades thymine- and uracil-containing DNA, if the enzyme can be completely inactivated, and whether it causes any downstream inhibition. To assess how efficiently Cod UNG degrades uracil-containing DNA, we generated a pool of 92 purified PCR products, where half contained thymine and half contained uracil. These were divided into eight groups including four to six different thymine-containing amplicons and equal number uracil-containing amplicons. Each group contained 1 to 16,384 target molecules per amplicon and reaction (Table S1). This PCR product pool was then used as template for preamplification using dUTP with either Cod UNG, heat inactivated Cod UNG, or water. All reactions were incubated for 5 min at room temperature, allowing Cod UNG to degrade uracil-containing template, before 20 cycles preamplification and downstream high-throughput qPCR (Figure 1). Four assays, three for thymine-containing amplicons and one for a uracil-containing amplicon, failed in the preamplification or following high-throughput qPCR, and were excluded from further analysis (Table S1). Active and inactivated Cod UNG, as well as water controls, displayed similar preamplification yield of thymine-containing amplicons (Figure 3A), indicating that Cod UNG neither degrades thymine-containing template nor inhibits downstream reactions. Heat inactivated Cod UNG treated samples also performed equally well when compared to controls that assessed the uracil-containing amplicons. In contrast, sample treatment with active Cod UNG completely removed all uracil-containing standards in all replicates for 34 of 45 assays (see Figure 3B and Table S1). In five of the remaining eleven assays, only one 1 of 15 replicates was positive for respective DNA assay. In the other six assays, ≥ 3 replicates were positive. Only 1 assay, *E2F1* (Table S1), the shortest uracil-containing assay included in the study, was positive for all replicates. Still, in average 97% of all uracil-containing template was degraded prior to preamplification. Analysis of all uracil-containing amplicons displayed a positive correlation between fraction of positive replicates and number of loaded DNA molecules per uracil (Figure 3C, Spearman’s correlation coefficient = 0.481, *p* < 0.001). Consequently, assays that are contaminated with many molecules and contain few uraciles in their sequences are the ones that still may suffer from carry-over contamination after Cod UNG treatment. These data show that Cod UNG can be completely and irreversibly inactivated and that the enzyme can be used to remove uracil-containing DNA molecules without inhibiting downstream amplification.

### 2.3. Single-Cell Gene Expression Profiling Using Preamplification with dUTP and Cod UNG

**Figure 4:**
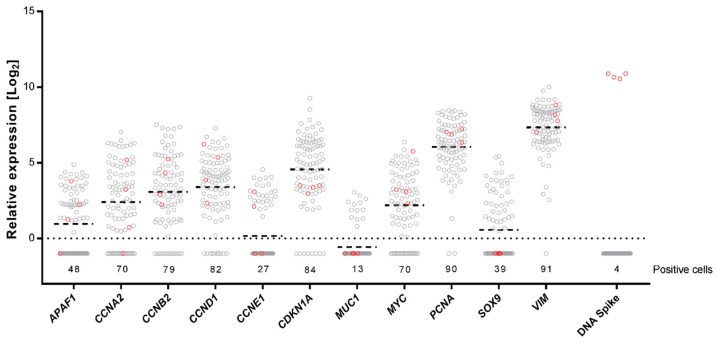
Single-cell gene expression profiling, where 92 single cells were treated with Cod uracil-DNA N-glycosylase (Cod UNG) in presence of 100 copies of an artificial uracil-containing DNA spike followed by preamplification using dUTP. The relative expression is shown for each gene where individual cells are represented by circles. The number of cells expressing each gene is indicated. Horizontal bars indicate mean expressions; the dotted line indicates one molecule. Four of the 92 cells were treated with heat inactivated Cod UNG (red), while the remaining 88 cells were treated with active Cod UNG (grey).

To demonstrate the use of Cod UNG treatment prior to target-specific preamplification, we performed single-cell gene expression profiling. We collected 92 human myxoid liposarcoma 402-91 cells in direct lysis buffer using fluorescence-activated cell sorting and subsequently reverse transcribed all of the RNA. Complementary DNA was treated with Cod UNG (*n* = 88), or heat inactivated Cod UNG (*n* = 4), prior to preamplification with dUTP, in the presence of a uracil-containing artificial DNA spike, added to mimic a contaminating DNA amplicon. Out of the 97 preamplified targets, including 96 different gene targets and one artificial DNA spike, we quantified the expression of 11 genes with qPCR. Selected genes were lowly, intermediately, and highly expressed (Figure 4). As expected, low transcription levels correlated with the low number of positive cells [22,23]. Quantification of the artificial DNA spike showed that Cod UNG eliminated all uracil-containing template in the 88 preamplification reactions, where the enzyme was active. On the contrary, high yield was evident in the four reactions where Cod UNG had been heat inactivated. This demonstrates that, with the addition of Cod UNG and the replacement of dTTP with dUTP in the preamplification, an accurate quantification of multiple rare target sequences in a limited sample size was possible, even in the presence of PCR generated contamination. 

## 3. Discussion

Biological and clinical analyses are increasingly dependent on accurate detection and quantification of DNA and cDNA biomarkers and are therefore sensitive to contamination, which can fabricate false-positives and inaccurate quantification. Analyses of multiple non-abundant nucleic acid biomarkers in limited sample sizes require preamplification. These studies are especially sensitive to sample-to-sample contamination since handling of large molecule numbers after preamplification is required before final analysis. Contamination can originate from three sources: (i) other test samples, (ii) experimental reagents and materials, and (iii) PCR products generated in previous amplification steps, i.e., carry-over contamination [24]. The risk of contaminations can be minimized by different means. For example, carry-over contamination can be reduced by following good laboratory practice. This involves physical separation of the individual experimental steps, where locations and equipment used in sample preparation, reaction setup, template loading, and post-PCR, are kept strictly apart. If material and equipment needs to be transferred, it should only be downstream in the experimental workflow, never in the reverse direction. Frequent change of gloves, use of laminar flow hoods, and disposable and DNA-free filter tips, reagents and plastics also minimize the likelihood of contamination. However, if contamination does occur, it needs to be eliminated [25]. This can be achieved by irradiation, enzymatic treatment, and the use of various chemicals [26]. 

Here, we have demonstrated the feasibility to perform target-specific preamplification with dUTP in the presence of Cod UNG in order to remove PCR generated contamination using DNA standards and single-cell gene expression profiling. While the enzyme did not succeed in complete degradation of all uracil-containing template, particularly for highly abundant amplicons with few uraciles, it did eliminate all amplicons for a majority of the assays. Therefore, Cod UNG should sufficiently degrade all uracil-containing amplicons from all but severe contaminations. Contaminations at these magnitudes are highly unlikely to occur following good laboratory practice, as discussed above. The Cod UNG degrading efficiency could potentially be optimized by increased enzyme concentration, temperature and/or time during which the template is incubated with Cod UNG [27], but that has not been evaluated here.

## 4. Materials and Methods

### 4.1. DNA Standards

Two types of DNA standards were used: one containing thymine and one containing uracil. The 96 thymine-containing DNA standards were generated as previously described [17]. Briefly, cDNA was used as a template in qPCRs to generate specific purified PCR products. DNA concentration was determined with a Qubit Fluorometer (Invitrogen, Carlsbad, CA, USA) and converted to molecules per microliter. Each uracil-containing DNA standard was generated using a pool containing 100 molecules, using all 96 thymine-containing DNA standards as template, or solely 100 copies of Universal DNA Spike I (TATAA Biocenter, Gothenburg, Sweden) in 15 µL qPCR reactions containing 1X Custom PreAmp Master Mix containing neither UNG nor dNTPs (Applied Biosystems, Foster City, CA, USA), 400 µM dATP, dCTP, dGTP, and 1200 µM dUTP (Roche Applied Science, Penzberg, Germany), 1X SYBR Green I (Invitrogen), and 400 nM mix of forward and reverse primers (Sigma-Aldrich, St. Louis, MO, USA, and TATAA Biocenter). Detailed primer information is provided in Table S1. Quantitative PCRs that generated uracil-containing PCR products were tested in 384-well plates (4titude, Wotton, UK), applying the following thermal protocol: 95 °C for 10 min, 45 cycles of 95 °C for 15 s, 60 °C for 60 s, followed by melt curve analysis:65 °C to 95 °C at 0.5 °C, per 5 s on a CFX384 Touch Real-Time PCR Detection System (Bio-Rad, Hercules, CA, USA). Correct PCR product formation for each assay was verified with a melt curve analysis and agarose gel electrophoresis. Each specific PCR product was purified with a MinElute PCR purification Kit and eluted in EB buffer (both Qiagen, Hilden, Germany). DNA concentration was quantified with the Qubit dsDNA HS Assay Kit (Invitrogen) on a Qubit Fluorometer and converted to molecules per microliter [28], and finally adjusted to 10^10^ molecules per microliter in nuclease-free water (Invitrogen) and stored at −20 °C.

### 4.2. Target-Specific Preamplification

Target-specific preamplification of purified DNA standards was executed in 10 µL reactions, which contained 1X Custom PreAmp Master Mix containing neither UNG nor dNTPs., 40 nM of each primer (all Sigma-Aldrich), 400 µM dATP, dCTP, dGTP, and either dTTP or dUTP (all Roche Applied Science), in addition to a 2 µL template. Ninety-six assays were used, where target-specific preamplification and downstream qPCRs were performed with identical primer pairs (Table S1). The following thermal profile was applied: 95 °C for 10 min, 20 cycles of 95 °C for 20 s, 60 °C for 4 min, followed by a final step at 60 °C for 10 min.

During the final elongation, step samples were moved immediately to dry ice. The samples were then slowly thawed on the ice, diluted 1:20 in 10 mM Tris, 1 mM EDTA, pH 8.0 (Invitrogen), and stored at −20 °C until analysis. For assessment of amplification efficiency, reproducibility, and sensitivity, the preamplification reactions were performed in 384-well plates on a CFX384 Touch Real-Time PCR Detection System. To analyze the effect of Cod UNG, the preamplification reactions were performed in 96-well plates (Sarstedt, Nümbrecht, Germany) on a T100 Thermal Cycler (Bio-Rad).

### 4.3. Cod UNG Treatment

Cod UNG treatment was performed by adding 0.01 U Cod UNG (ArcticZymes, Tromsø, Norway) per microliter final reaction volume, followed by an incubation at room temperature for 5 min prior to target-specific preamplication. For assessment of heat inactivated Cod UNG, the enzyme was incubated at 95 °C for 2 min.

### 4.4. High-Throughput Quantitative Real-Time PCR

High-throughput qPCR was performed on the BioMark system (Fluidigm, South San Francisco, CA, USA), using the 96.96 Dynamic Array Chip for Gene Expression (Fluidigm). Each 6 μL sample reaction contained 1.5 µL diluted preamplification product as template, 3.3 µL 2X TATAA EvaGreen GrandMaster Mix Low Rox (TATAA Biocenter), 0.3 µL 20X DNA Binding Dye Sample Loading Reagent (Fluidigm), 0.33 µL 20X EvaGreen (Biotium, Fremont, CA, USA), and 0.57 µL water. The 6 μL assay reaction mix contained 3 µL of Assay Loading Reagent (Fluidigm), 2.7 µL of 10 µM mix of reverse and forward primers, and 0.3 µL of water. Priming and loading of the dynamic array was performed according to the manufacturer’s instructions. The temperature profile was: thermal mixing at 50 °C for 2 min, 70 °C for 30 min, and 25 °C for 10 min, followed by polymerase activation at 95 °C for 60 s and 40 cycles of amplification; 95 °C for 10 s, 60 °C for 20 s, and 72 °C for 20 s. The melt curve analysis was performed in the range of 65 to 98 °C at 1 °C, per 3 s. Amplification data were analyzed with the Fluidigm Real-Time PCR Analysis software (ver. 4.1.3, Fluidigm), applying the linear derivative baseline subtraction method and a user-defined global threshold to obtain cycle of quantification (Cq) values. Melt curve analysis was performed on all samples in order to eliminate samples with non-specific PCR products. All high-throughput qPCR experiments were conducted in accordance with the Minimum Information for Publication of Quantitative Real-Time PCR Experiments guidelines [29].

Individual assay amplification efficiencies and intercepts were determined using standard curves in triplicate (5120, 1280, 320, 80, 20, and 5 molecules per reaction) for amplification using dTTP and dUTP, respectively. Outliers were identified and removed by Grubbs’ test using GenEx (version 6.1.0.757, MultiD, Gothenburg, Sweden). The individual assay amplification efficiencies and intercepts were subsequently used to calculate number of molecules per sample and log_2_-transformed. Missing data and outliers were replaced by the average of remaining replicates. For standard samples with ≤5 molecules, missing data and outliers were replaced with 0.5X the minimum value of remaining replicates. For the Cod UNG treated dUTP-containing assays, all missing data and outliers were replaced with 0.5 copies. The rationale for handling missing data are the following: at >5 molecules missing data and outliers are likely due to technical failures, whereas at ≤5 molecules missing data and outliers are likely due to no molecules present in the reaction [1,30]. Sensitivity was calculated as the percentage of reactions loaded with 5 molecules, which were positive for correct product formation and after outlier removal.

### 4.5. Single-Cell Quantitative Real-Time PCR

Human myxoid liposarcoma cell line 402-91 was cultured in RPMI 1640 GlutaMAX medium supplemented with 5% fetal bovine serum, 50 U/mL penicillin and 50 mg/mL streptomycin (all Gibco, Grand Island, NY, USA) at 37 °C and 5% CO_2_. Cells were washed with 1X PBS, pH 7.4, supplemented with 3 mM EDTA, and enzymatically dissociated with 0.25% Trypsin supplemented with 0.5 mM EDTA (all Gibco). Cells were then resuspended in 1X PBS, pH 7.4 (Gibco), supplemented with 2% bovine serum albumin (Sigma-Aldrich), and kept on ice until sorting. Cell aggregates were removed by filtering through a 70 µm cell strainer (BD Biosciences, Franklin Lakes, NJ, USA). Individual cells were then sorted into 96-well PCR plates, using a BD FACSAria II (BD Biosciences). Cells were sorted directly into 5 µL lysis buffer containing 1 mg/mL bovine serum albumin and 2.5% glycerol (Thermo Scientific, Waltham, MA, USA) [31]. The flow cytometry instrument was manually calibrated to deposit single cells in the center of each well and 7-Aminoactinomycin D (Sigma-Aldrich) was used as viability marker in the sorting procedure. Sorted plates were immediately frozen on dry ice and stored at −80 °C.

Single-cell cDNA synthesis was performed on 92 individual cells using TATAA GrandScript cDNA Synthesis Kit (TATAA Biocenter). Briefly, 2 µL 5X TATAA GrandScript RT Reaction Mix, 0.5 µL TATAA GrandScript RT Enzyme, and 2.5 µL nuclease-free water were added to the lysed cells for a final volume of 10 µL. The following thermal program was used: 22 °C for 5 min, 42 °C for 30 min, 85 °C for 5 min on a T100 Thermal Cycler, and samples were stored at −20 °C.

Target-specific preamplification of single-cell cDNA was carried out in 50 µL reactions on a T100 Thermal Cycler in 96-well plates containing 1X Custom PreAmp Master Mix containing neither UNG nor dNTPs, 40 nM of each primer, 400 µM dATP, dCTP, dGTP and dUTP, either 0.5 U Cod UNG, or 0.5 U heat inactivated Cod UNG, 100 molecules uracil-containing Universal DNA Spike I and 5 µL cDNA. The primer pool contained all 97 primer pairs (Table S1) including the primers for Universal DNA Spike I. The same protocols for thermal cycling, dilution and storage was applied as described above for DNA standards. 

Single-cell qPCR was performed in 6 μL reactions containing 1X TATAA SYBR GrandMaster Mix (TATAA Biocenter), 400 nM of forward and reverse primers (Table S1), and 2 μL preamplified cDNA. Quantitative PCRs were carried out in 384-well plates applying the following thermal protocol: 95 °C for 2 min, 35 cycles of 95 °C for 5 s, 60 °C for 20 s, 70 °C for 20 s, followed by a melt curve analysis: 65 °C to 95 °C at 0.5 °C per 5 s on a CFX384 Touch Real-Time PCR Detection System. Melt curve analysis was performed on all samples to eliminate any samples with non-specific PCR products. The cycle of quantification value was determined by the second derivative maximum method. All single-cell qPCR experiments were conducted in accordance with the Minimum Information for Publication of Quantitative Real-Time PCR Experiments guidelines [2,29]. Single-cell data were pre-processed using GenEx, as previously described [30]. Briefly, a Cq-value of 24 was chosen as cut-off value and missing data were replaced with Cq-value 25. Cq-values were converted to relative quantities assuming 100% PCR efficiency and log_2_-transformed.

### 4.6. Statistical Analyses

All statistical tests, except Grubbs’ test, were performed using the GraphPad Prism software (version 7.02, GraphPad Software, La Jolla, CA, USA) and were considered significant if *p* < 0.05.

## 5. Conclusions

In conclusion, we show that, prior to targeted preamplification with dUTP, the use of Cod UNG efficiently removes PCR generated contamination. Cod UNG is completely compatible with downstream preamplification and can be completely inactivated, a prerequisite for successful quantification, as any loss of amplicons results in quantification biases. We also show that using dUTP instead of dTTP in the preamplification reaction yields similar sensitivity, possibly improved reproducibility, but somewhat lower amplification efficiency. If needed, decreased amplification efficiency can be compensated by additional cycle(s) of preamplication [18]. Preamplification generated contamination is a concern in all clinical and biological applications, but with good laboratory practice and preamplification using dUTP in the presence of Cod UNG, the problems can be minimized.

## Supplementary Materials

The following are available online at http://www.mdpi.com/1422-0067/19/10/3185/s1.
