# Supplementary material for: Preamplification with dUTP and Cod UNG Enables Elimination of Contaminating Amplicons
Source: Int J Mol Sci. 2018 Oct 16;19(10):3185. doi: 10.3390/ijms19103185 (PMC6214100; doi:10.3390/ijms19103185)
Supplement: Supplementary file 1 [file ijms-19-03185-s001.pdf]

Supplementary Table S1: Detailed qPCR assay information.

Gene	Ref Seq.	Forward primer [5'-3']<br>Reverse primer [5'-3']	Amplicon length [bp]<br>Bp between primers	[1] n_T or n_U	[2] PCR E_{dTTP}<br>[3] PCR E_{dUTP}	[4] Molecules/reaction<br>[5] T or U
APAF1	NM_013229.2	ATGGAACAGTGAAGGTATGGAA<br>AGCAGAGGTAGATGAAAACTTGGT	125<br>79	51	100.8%<br>94.1%	1<br>U
ATM	NM_000051.3	CCGTCAGCAAAGAAGTAGAAGGA<br>GCTAAGTGTGGGATAGAGCGA	182<br>138	86	109.2%<br>97.7%	1<br>U
ATR	NM_001184.3	GGATGCCACTGCTTGTTATGA<br>TCCACTCGGACCTGTTAGC	152<br>112	63	105.5%<br>97.6%	1<br>U
BBC3	NM_001127240.2	TACGAGCGGCGGAGACAAG<br>CTGGGTAAGGGCAGGAGTC	95<br>57	21	109.2%<br>101.7%	4096<br>T
BCL2L1	NM_138578.2	TGCGTGGAAAGCGTAGACAA<br>ACAAAAGTATCCCAGCCGCC	125<br>85	39	117.8%<br>94.5%	1<br>T
BID	NM_197966.2	GCCAGAAATGGGATGGACTGA<br>TCTCTAGGAACGCTGTTGACAT	127<br>84	38	102.4%<br>94.0%	4096<br>T
CASP3	NM_004346.3	GGCGTGTCATAAAATACCAGTG<br>GCGTCAAAGGAAAAGGACTCAAA	216<br>171	91	106.4%<br>89.9%	1024<br>U
CAV1	NM_001753.4	GCAACATCTACAAGCCCAACA<br>GCCTTCCAAATGCCGTCAAAA	196<br>154	72	96.3%<br>91.8%	1024<br>U
CCNA2	NM_001237.3	AAGACGAGACGGGTTGC<br>GGCTGTTTACTGTTTGCTTTCC	89<br>50	27	102.5%<br>95.4%	1<br>T
CCNB2	NM_004701.3	CGACCCTTGCCACTACACTT<br>TGACTTCCAATACTTCATTCTCTG	244<br>200	111	101.7%<br>95.7%	1<br>U
CCND1	NM_053056.2	AGAGGCGGAGGAGAACAAA<br>TGAGGCGGTAGTAGGACAGG	182<br>143	58	101.5%<br>93.7%	1<br>T
CCND3	NM_001760.4	CTGTTCGCTGCCCGAGTATG<br>GCATGTGCGGCTTGATCTC	186<br>147	38	94.1%<br>89.8%	[7] N.I.<br>[7] N.I.
CCNE1	NM_001238.3	CCGGTATATGGCGACACAAG<br>TACGCAAACTGGTGCAACTT	123<br>83	57	98.1%<br>94.0%	1<br>U
CCNG1	NM_004060.3	CCTTGGGTGTGTTGGACTGA<br>GCTATTTCTCCTTTCAAGTGGCA	250<br>207	129	101.8%<br>88.4%	1<br>U
CCNH	NM_001239.3	TGAGGAGGAAGAATGGACTGA<br>GTTACTGTGAAAGGGAAAGAAAACA	189<br>143	104	107.9%<br>107.4%	1<br>T
CDC25A	NM_001789.2	GTCGCCTGTCACCAACCT<br>CGGAGGAGCCCATTCTCT	116<br>80	38	106.6%<br>90.4%	1<br>T
CDC25B	NM_021873.3	GGCGGGCACATCAAGACTG<br>GGGTAGTAGAGGCTGGGGTA	209<br>170	71	[6] -	4096<br>T
CDC45	NM_001178010.2	CTTTGAGTATGACCTCCGCCT<br>AACTTCTGCTTCACCTGCTTCA	174<br>131	54	131.6%<br>120.4%	1<br>T
CDC6	NM_001254.3	ACTTGGTGCTGATTGGTATTGC<br>TGCGAACATCTCCTGAAACA	237<br>195	113	93.7%<br>89.2%	4<br>U
CDC7	NM_003503.3	ACACCAGGATTCAGAGCACC<br>TCGTCCACTAAGCAAAGAAAGAA	108<br>65	36	95.3%<br>98.3%	4<br>U
CDK1	NM_001786.4	AGAAAGTGAAGAGGAAGGGGTTC<br>AGCACATCCTGAAGACTGACTA	99<br>54	35	97.6%<br>87.9%	4<br>U
CDK6	NM_001259.7	CGTGGTCAGGTTGTTTGATGT<br>CGGTGTGAATGAAGAAAGTCC	195<br>153	86	97.6%<br>88.1%	4096<br>T
CDK7	NM_001799.3	GCCAGAGATAAGAACACCAACC<br>TCCAAAAGCATCAAGGAGACCA	162<br>118	81	93.4%<br>100.7%	4<br>U
CDKN1A	NM_000389.4	TTAGCAGCGGAACAAGGAGT<br>GCCGAGAGAAAACAGTCCAG	225<br>185	87	99.1%<br>93.7%	4<br>U
CDKN1B	NM_004064.4	TCTGAGGACACGCATTTGGT<br>CTTAATTCGAGCTGTTTACGTTTGA	240<br>195	91	107.5%<br>94.5%	4<br>T
CDKN2C	NM_001262.2	AGTTCCTGGTGAAGCACACGG<br>CCTCATTCCTCCCATAGAGCCT	96<br>53	21	119.2%<br>98.5%	4<br>T
CDT1	NM_030928.3	GACTCGTGCTGCCCTACAA<br>ACTCCTCAAAACGCCTACG	153<br>115	42	94.9%<br>88.6%	1024<br>U
COL1A1	NM_000088.3	CGGAGCAGACGGGAGTTTC<br>TGTACGCAGGTGATTGGTGG	247<br>208	78	105.5%<br>92.7%	4096<br>T
DBF4	NM_006716.3	GCCAGAAAAATCCAAATGTAAGCC<br>TTCAACTCGCCCTCCCAGAT	118<br>74	48	103.4%<br>92.1%	4<br>U
E2F1	NM_005225.2	TGCCAAGAAGTCCAAGAACCA<br>GTCAACCCCTCAAGCCGTC	81<br>41	12	121.4%<br>89.2%	16384<br>U
E2F4	NM_001950.3	CGGACCCAACCCTTCTACCT<br>GGGGCAAACACTTCTGAGGA	156<br>116	54	[6] -	[7] N.I.<br>[7] N.I.
E2F5	NM_001951.3	TGATACTTTGGCTGTGAGGCA<br>CAGCACCTACACCTTTCCAC	119<br>78	55	106.3%<br>103.1%	4<br>T
E2F7	NM_203394.2	CCTTTAGCCCACCCAGTATTT<br>ATCCCTCTCTGACCCTGACC	237<br>196	86	96.8%<br>86.9%	1024<br>U
EGFR	NM_005228.4	AGAGCGACTGCCTGGTCT<br>TACGGGGACACTTCTTCACG	159<br>121	50	94.6%<br>92.3%	4096<br>T
EWSR1	NM_013986.3	ATACTACTCCAACTGCCCCC<br>TCCATCCTGCGGTCTTGTA	197<br>158	65	95.1%<br>93.8%	1024<br>T

FN1	NM_212482.2	CTGAGGGCAGAAGAGACAACA<br>CCACGACCATTCCCAACACA	205<br>164	77	105.3%<br>86.8%	16384<br>U
GTSE1	NM_016426.6	TGCGGAGAAGCCCAAGAAAGAG<br>TGCGAGATTGCTGGTAGAGCC	229<br>186	78	89.5%<br>101.5%	4<br>T
HIF1A	NM_001530.3	CGATTTTGGCAGCAACGACACA<br>CGTTTCAGCGGTGGGTAATGGA	142<br>120	74	107.0%<br>91.8%	1024<br>T
ID1	NM_002165.3	CTGAGGGAGAACAAGACCGAT<br>CCCCCTAAAGTCTCTGGTGA	132<br>91	30	106.6%<br>90.0%	1024<br>T
IFNAR1	NM_000629.2	GTGAGAAAACAAAACCAGGAAATAC<br>TGACAAACGGGAGAGCAAAT	84<br>39	28	99.8%<br>102.2%	4<br>T
IFNAR2	NM_207585.2	GGATTCAGCGGGAACACA<br>CCTTTTATTTCGGGTTTATGCTTCT	227<br>184	114	[6] -	4<br>T
IFNGR1	NM_000416.2	TCCAGTTGTTGCTGCTTTACT<br>AACGGCTCTTCACAGACCAC	231<br>190	125	104.2%<br>84.2%	16<br>U
IFNGR2	NM_001329128.1	TTTTCGTTGCTGTCGGTGC<br>TGGGCTGAGTTGGGTCTTTT	139<br>100	57	103.7%<br>91.0%	16<br>U
IGF2BP3	NM_006547.2	TCCCAAAAAGGCAAAGGATTCG<br>GCTCTCCACCACTCCATACTG	107<br>64	36	108.3%<br>96.1%	16<br>U
IL6ST	NM_002184.3	GAGGTGTGAGTGGGATGGTG<br>GCGGATTGGGCTTCACTTTA	248<br>208	124	91.8%<br>95.0%	16<br>U
IRF9	NM_006084.4	CCATCAAAGCGACAGCACAG<br>GCCCCCTCCTCCTCATTATT	122<br>82	36	96.6%<br>95.7%	1024<br>U
JAK1	NM_002227.3	CTGGAGTATCTGTTTGCTCAGG<br>GCTCGGTCTTGGGGTCTC	76<br>36	20	[6] -	16<br>T
LOX	NM_002317.6	CCAGTACAGCATACAGGGCA<br>TGGCATCAAGCAGGTCATAG	192<br>152	84	97.3%<br>90.5%	16384<br>T
MCM10	NM_182751.2	CTTCTCTGGTCTGCGGCTC<br>AGGTTTTTCCACTATTCACACTCT	197<br>154	83	101.9%<br>97.8%	16<br>U
MCM4	NM_005914.4	GCCAAACGCCTCCATCG<br>GGCACTCATCCCCGTAGTAAG	96<br>58	28	105.5%<br>92.9%	16<br>U
MCM5	NM_006739.	ACTTCACCAAGCAGAAATACCCG<br>GGCAGAGGTCCCAGCAACAT	206<br>163	56	97.2%<br>93.7%	16<br>T
MCM6	NM_005915.5	AGCGGAACTTTTCTGTGCTT<br>CTTCTAAACTGCGGGGGATAC	220<br>179	102	96.6%<br>85.4%	16<br>T
MCM7	NM_005916.4	AACTGTGCGTGGAATCGTCA<br>GAGACTGGATCGGCTGGTAG	110<br>70	32	105.0%<br>95.1%	16<br>T
MCM8	NM_032485.5	TCTTCCCACAAAGTGTCCTGT<br>CCGACCTGCTTCTCTCTGAT	142<br>101	50	96.8%<br>96.8%	16<br>T
MCM9	NM_017696.2	AGGCTGGGGCATTAGTTCTT<br>ATGGTGGTCCTTGTGTTCAG	163<br>123	64	98.2%<br>99.6%	16<br>T
MDM2	NM_002392.5	ATCAGCAGGAATCATCGGAC<br>GTGGCGTTTTCTTTGTCGTT	221<br>181	105	100.2%<br>87.6%	64<br>U
MDM4	NM_002393.4	TGCCGCTTTTGAAGATTTTGC<br>GAGAGGGCTTGGGTCTTTCA	203<br>162	89	97.6%<br>97.7%	64<br>U
MMP2	NM_004530.5	GTCCGTGTGAAGTATGGGAAC<br>CCCTGGAAGCGGAATGGAAAC	236<br>194	84	103.0%<br>95.8%	16384<br>T
MUC1	NM_002456.5	CTGGTCTGTGTTCTGGTTGC<br>CCACTGCTGGGTTTGTGTAA	250<br>210	90	104.2%<br>88.4%	1024<br>T
MYC	NM_002467.5	GGAGGCTATTCTGCCCATTT<br>GGCTGCTGGTTTTCCACTAC	122<br>82	33	108.2%<br>93.3%	1024<br>T
ORC1	NM_004153.3	CAAGCCTAGAACGCCACG<br>TACATGCACCCTCCGGTATG	207<br>169	77	109.1%<br>99.7%	64<br>U
ORC2	NM_006190.4	ACCTAGCGGTGACTGTATCTG<br>CTCCCACAAAGTGAACCTCCA	122<br>80	50	101.4%<br>92.6%	16384<br>U
ORC4	NM_181741.3	ACACATGCTATTGATGCTTGCT<br>TGCTACACAGTTGGCTTGCT	95<br>53	31	103.7%<br>96.6%	64<br>U
ORC5	NM_002553.3	TGCCAGAATGCCCCACTT<br>TGATGTCTCTCTCCAAACAAGGA	87<br>46	22	106.7%<br>91.8%	64<br>U
ORC6	NM_014321.3	GAAGCCCCAGCAAAGGAAATG<br>AAATCCCAAAGCCGTCAAGT	223<br>182	108	96.2%<br>89.7%	1024<br>U
OSMR	NM_003999.2	TGTCATCTGGGTGGGGAAT<br>CTCAGGGAACTTGGCATCGT	142<br>103	54	95.7%<br>92.9%	64<br>U
PCNA	NM_002592.2	GTGGAGAACTTGGAAATGGAA<br>ACCGTTGAAGAGAGTGGAGTG	160<br>118	74	96.2%<br>98.0%	64<br>T
PDGFA	NM_002607.5	CACCACCGCAGCGTCAA<br>CCGTGTCCTCTTCCCGATAA	145<br>108	51	102.9%<br>94.1%	1024<br>T
PIAS1	NM_001320687.1	ACCTGTCCTTCCCTATCTCCC<br>GGTGTTGTAATGCTGATTGTCTCC	237<br>192	111	106.4%<br>97.7%	64<br>T
PIAS3	NM_006099.3	GAGCCGACATCCAAGGTTTAG<br>CCAGAAAGTGAGAAGGGGTCC	144<br>102	54	106.2%<br>101.0%	64<br>T
PPARG	NM_138712.3	TACTCCACATTACGAAGACAT<br>CTCCATAGTGAAATCCAGAAG	236<br>194	110	97.1%<br>97.1%	4096<br>U
PPM1D	NM_003620.3	AAGGGTTTCACCTCGTCCG<br>GCCATTCCGCCAGTTTCTTC	100<br>61	26	111.9%<br>103.0%	64<br>T

RB1	NM_000321.2	AAAGGACCGAGAAGGACCA<br>AAGGCTGAGGTTGCTTGTGT	180<br>141	85	103.4%<br>99.6%	64<br>T
RCHY1	NM_015436.3	ACTGTGGAATTTGTAGGATTGGT<br>ACATGAGCAACAACACGGGA	172<br>129	82	102.2%<br>98.5%	64<br>T
RPS10	NM_001014.5	AGCCGCAGAGATGTTGATG<br>CCTCGGGACTTGAGAGACTG	177<br>138	69	91.6%<br>89.3%	4096<br>U
RRM2B	NM_015713.4	TGTGACTTTGCTTGCCTGATG<br>TGCCTGAAAAACCTTTGAGAATCC	222<br>177	109	102.5%<br>92.1%	256<br>U
SESN1	NM_014454.2	GGGAGTGAAGACGCACAGAT<br>GCCGCAGCCATTATTCCAA	197<br>158	95	109.6%<br>94.0%	256<br>U
SHISA5	NM_016479.4	GTGGTGAGGTGTGTATGGCTT<br>AGGTCGCTCCGAACCCTGA	249<br>209	84	104.0%<br>93.1%	256<br>U
SIAH1	NM_003031.3	CGCTCTCCGCCCACAGAAAT<br>GGACACTCAAAAAGACTCGCCA	143<br>101	44	104.7%<br>90.2%	256<br>T
SKP2	NM_005983.3	CCCCAGGAACTGCTCTCAAA<br>ACTCATCAGACGCTAGGCGA	250<br>210	97	100.4%<br>93.2%	256<br>U
SOCS2	NM_003877.4	CATGACCCTGCGGTGCCTT<br>AAGTTCCTTCTGGTGCCTCTTTT	209<br>167	57	67.1%<br>69.9%	[7] N.I.<br>[7] N.I.
SORT1	NM_002959.6	ATGGGAAGAAATCCACAAAGCAG<br>ATTCCAGAGCCCCAAGGTCAG	113<br>69	39	101.0%<br>92.6%	4096<br>U
SOX9	NM_000346.3	GCTCTGGAGACTTCTGAACGA<br>CCGTTCTTCACCGACTTCCT	132<br>91	29	98.3%<br>94.0%	4096<br>U
STAT1	NM_139266.2	GTTATGGGACCGCACCTTCA<br>CACCAACAGTCTCAACTTCACAG	148<br>105	46	107.5%<br>92.1%	256<br>U
STAT2	NM_005419.3	AGCACCAGGATGATGACAAGG<br>GGGGGATTCGGGGATAGAGG	162<br>121	59	101.2%<br>84.3%	256<br>T
STAT3	NM_139276.2	GCCAGAGAGCCAGGAGCATC<br>GGGACATCGGCAGGTCAAT	119<br>80	37	105.8%<br>98.9%	256<br>U
STAT5B	NM_012448.3	CTGCGAGTCTGCTACTGCTA<br>GAGTCAGGGTTCTGTGGGTA	186<br>146	61	106.0%<br>94.7%	256<br>T
STAT6	NM_001178078.1	GAACATCCAGCCATTCTCTGC<br>TTGGTCCCTTTCCACGGTCA	202<br>161	76	96.8%<br>87.2%	256<br>T
SUZ12	NM_015355.3	AGCCATCACCAAACTCAGAAA<br>GCTTTTTACCTGTGGGAACTTG	126<br>83	52	[6] -	4096<br>U
TGFB1	NM_000660.6	AACAATTCCTGGCGATACCTCA<br>AAGCCCTCAATTTCCCCTCC	125<br>83	33	95.9%<br>89.9%	16384<br>U
TGFB2	NM_001135599.3	AAGACCCCACATCTCCTGCTAA<br>TCGTGTATCCATTTCCACCCT	182<br>139	74	96.2%<br>95.9%	16384<br>T
TGFB3	NM_003239.4	GCGTGAGTGGCTGTTGAGA<br>AGGATTAGATGAGGGTTGTGGTG	216<br>174	91	90.0%<br>92.2%	16384<br>T
TP53INP1	NM_033285.3	GCCCAAGTAGTCCCAGAGTG<br>AGTTGTATGAGCAGCAAGAGC	86<br>45	28	103.0%<br>93.9%	[7] N.I.<br>[7] N.I.
TYK2	NM_003331.4	AGCTTGTACTGCTACGATCCG<br>GACTTCTCGCCTTGGTCCTC	185<br>144	55	99.4%<br>92.7%	256<br>T
VIM	NM_003380.4	CAGATGCGTGAAATGGAAGA<br>TGGAAGAGGCAGAGAAATCC	222<br>182	92	110.1%<br>91.5%	4096<br>U
ZMAT3	NM_022470.3	TATCGAAGGGAGGGGAGCAA<br>TTAAAGGAGCCCATCTGCGG	247<br>207	105	108.0%<br>98.8%	256<br>T
TATAA Universal DNA Spike I	n/a	n/a<br>n/a	300<br>n/a	n/a	Not tested<br>Not tested	[7] N.I.<br>[7] N.I.

[1] *n_T or n_U*: Total number of thymidines or uraciles in sequence between primers.
[2] *PCR E_{dTTP}*: Amplification efficiency using dTTP in preamplification.
[3] *PCR E_{dUTP}*: Amplification efficiency using dUTP in preamplification.
[4] *Molecules/reaction*: Number of amplicons per reaction. Related to data in Figure 3.
[5] *T or U*: Whether amplicon contains thymine (T) or uracil (U). Related to data in Figure 3.
[6] -: Failed amplification.
[7] *N.I.*: Not included.
